# Supplementary material for: Elicitation of stakeholder viewpoints about medical cannabis research for pain management in critically ill ventilated patients: A Q-methodology study
Source: PLoS One. 2021 Mar 18;16(3):e0248475. doi: 10.1371/journal.pone.0248475 (PMC7971896; doi:10.1371/journal.pone.0248475)
Supplement: S1 Appendix — (DOCX) [file pone.0248475.s001.docx]

S1 Appendix

**Topic Statement**

*One common form of life support for severely ill patients admitted to an intensive care unit is mechanical ventilation, or sometimes referred to as a breathing machine. Most ventilated patients require treatment to endure the pain, anxiety and suffering that is commonly experienced by these* *patients. Usually, medications such as opioids and benzodiazepines are used in these patients to manage their symptoms. Both opioids and benzodiazepines may have side effects at the time of their use, including confusion, sedation, and loss of memory along with some other longer-term effects such as addiction or post-traumatic stress disorder. Cannabinoid medication, commonly known as Medical Cannabis, has been effectively used to alleviate pain, post-traumatic stress disorder and anxiety in patients in other settings with potentially fewer side effects and reduced risk of developing addiction. Some are suggesting that Medical Cannabis should be used in addition to opioids and benzodiazepines in ventilated patients to alleviate suffering and reduce the use of these other medications. However, there may be challenges, as well as concerns regarding safety, effectiveness, legalities or resistance to using Medical Cannabis for patients in this setting, or in general*. *Research demonstrating the benefits should be done before considering using Medical Cannabis in these patients.*

**Table 1**

| Number | Statement |
| --- | --- |
| 1 | I support MC research in critically ill patients that primarily aims to reduce the use of other harmful drugs such as opioids or benzodiazepines |
| 2 | I don't support MC research in critically ill patients because it encourages its use as a recreational drug |
| 3 | I support MC research in critically ill patients because the benefits of MC have been exaggerated compared to the evidence that supports its use |
| 4 | I don't support MC research in critically ill patients because it may prevent research into other non-pharmacological alternatives to alleviate pain and suffering in these patients |
| 5 | I support MC research in critically ill patients as long as it does not include vulnerable groups such as those with mental health disorders, pregnant females or children < 18 year olds |
| 6 | I don't support MC research in critically ill patients because opioids and benzodiazepines have been used for a long-time and shown to be safe in ICU patients |
| 7 | I support MC research in critically ill patients that is funded by cannabis-producing companies so long as they don't influence results of the study |
| 8 | I don't support MC research in critically ill patients because practical experience shows that cannabis is safe and may reduce the use of harmful drugs |
| 9 | I support research in MC use in critically ill patients, as long as the public and health care providers are better educated about the proper use and effects of MC at the same time |
| 10 | I don't support MC research in critically ill patients because cannabis should be available for use as it is a natural and legal product |
| 11 | I support MC research in critically ill patients using only plant-based and not synthetic MC |
| 12 | I don't support MC research in critically ill patients because the societal stigma of cannabis use may negatively affect the doctor-patient relationship |
| 13 | Given that there is a lack of evidence about the benefits of MC use in ICU patients, I support research to determine its potential benefits in these patients |
| 14 | I don't support MC research in critically ill patients because using MC in patients already prescribed opioids may increase their risk of addiction |
| 15 | I support MC research as long as it does not require smoking or vaporization |
| 16 | I don't support MC research in critically ill patients because it may increase the costs of health care |
| 17 | I support MC research in critically ill patients that uses MC that is THC-free |
| 18 | I don't support MC research in critically ill patients because the use of MC may mask signs of pain and suffering and result in under-treatment |
| 19 | I support MC research in critically ill patients because the societal stigma of cannabis may have undermined the potential benefits of MC use |
| 20 | I don't support MC research in critically ill patients that is funded by cannabis-producing companies |
| 21 | I support MC research in critically ill patients that primarily aims to reduce outcomes such as anxiety and pain during their ICU stay |
| 22 | I don't support MC research in critically ill patients because it is not feasible given all the possible formulations of CBD:THC in MC |
| 23 | I support MC research in critically ill patients that primarily aims to reduce long-term outcomes such as PTSD and chronic pain in ICU survivors |
| 24 | I don't support MC research in critically ill patients because we need better adherence to the recommended sedation guidelines for ventilated patients to prevent pain and suffering rather than adding another drug |
| 25 | I support MC research in any critically ill patient regardless if they are on a ventilator or not |

**Table 2**^1^.

| Qsort | Factor1 | Factor2 | Factor3 | Uniqueness |
| --- | --- | --- | --- | --- |
| qsort1 | 0.7835 | -0.0824 | 0.1149 | 0.3439 |
| qsort2 | 0.4931 | -0.6677 | -0.1086 | 0.6191 |
| qsort3 | 0.8678 | -0.1545 | 0.1609 | 0.2009 |
| qsort4 | 0.4544 | 0.2989 | 0.3339 | 0.2525 |
| qsort5 | 0.0992 | 0.4502 | 0.3203 | 0.5225 |

^1^ Uniqueness = total amount of variance not accounted for by the factors, and this includes both measurement and specific error variance. Using a p<0.05, factor loads were considered significant when values ≥ |0.39|.

*Interpretation: Using q-sort 1 as an example, the uniqueness is 34.39% so the total variance explained by the 3 factors = (0.78)^2^ + (-0.08)^2^ + (0.115)^2^ = 0.633 (= 63.3% = (1 – uniqueness)). The analogy to a regression model is as follows:*

*(q-sort pattern)_i_ = (factor loading)_i_*factor_i_ + uniqueness_i_  where:*

*q-sort pattern = observed outcome variable Y_i_ (Dependent variable)*

*Factor loading = regression coefficient ß_i_*

*Factor = Independent variable X_i_*

*Uniqueness = residual error e_i_*

**Table 3**

| Statements | | Rank | | |
| --- | --- | --- | --- | --- |
| **Factor 1** | | Factor 1 | Factor 2 | Factor 3 |
| 21 | I support MC research in critically ill patients that primarily aims to reduce outcomes such as anxiety and pain during their ICU stay | 4 | 1 | 0 |
| 23 | I support MC research in critically ill patients that primarily aims to reduce long-term outcomes such as PTSD and chronic pain in ICU survivors | 3 | 1 | 0 |
| 19 | I support MC research in critically ill patients because the societal stigma of cannabis may have undermined the potential benefits of MC use | 2 | 0 | 0 |
| 25 | I support MC research in any critically ill patient regardless if they are on a ventilator or not | 2 | -3 | -1 |
| 3 | I support MC research in critically ill patients because the benefits of MC have been exaggerated compared to the evidence that supports its use | 0 | 2 | -2 |
| 5 | I support MC research in critically ill patients as long as it does not include vulnerable groups such as those with mental health disorders, pregnant females or children < 18 year olds | 0 | 4 | 4 |
| **Factor 2** | |  |  |  |
| 7 | I support MC research in critically ill patients that is funded by cannabis-producing companies so long as they don't influence results of the study | 1 | 2 | -1 |
| 2 | I don't support MC research in critically ill patients because it encourages its use as a recreational drug | -4 | 0 | -3 |
| 4 | I don't support MC research in critically ill patients because it may prevent research into other non-pharmacological alternatives to alleviate pain and suffering in these patients | -3 | 0 | -1 |
| **Factor 3** | |  |  |  |
| 24 | I don't support MC research in critically ill patients because we need better adherence to the recommended sedation guidelines for ventilated patients to prevent pain and suffering rather than adding another drug | -2 | -4 | 2 |
| 20 | I don't support MC research in critically ill patients that is funded by cannabis-producing companies | 0 | -2 | 2 |
| 1 | I support MC research in critically ill patients that primarily aims to reduce the use of other harmful drugs such as opioids or benzodiazepines | 3 | 3 | 1 |
| 18 | I don't support MC research in critically ill patients because the use of MC may mask signs of pain and suffering and result in under-treatment | -1 | -1 | 1 |

**Table 4**

| Statements | | Rank | | |
| --- | --- | --- | --- | --- |
|  |  | Factor 1 | Factor 2 | Factor 3 |
| 9 | I support research in MC use in critically ill patients, as long as the public and health care providers are better educated about the proper use and effects of MC at the same time | 1 | 3 | 3 |
| 13 | Given that there is a lack of evidence about the benefits of MC use in ICU patients, I support research to determine its potential benefits in these patients | 2 | 2 | 1 |
| 11 | I support MC research in critically ill patients using only plant-based and not synthetic MC | 0 | 1 | 1 |
| 14 | I don't support MC research in critically ill patients because using MC in patients already prescribed opioids may increase their risk of addiction | -1 | -1 | -1 |
| 10 | I don't support MC research in critically ill patients because cannabis should be available for use as it is a natural and legal product | -1 | 0 | -2 |
| 12 | I don't support MC research in critically ill patients because the societal stigma of cannabis use may negatively affect the doctor-patient relationship | -1 | -2 | -2 |
| 8 | I don't support MC research in critically ill patients because practical experience shows that cannabis is safe and may reduce the use of harmful drugs | -2 | -1 | -3 |

**Table 5**

| Factor | Label | Description | Example of Supportive Statement |
| --- | --- | --- | --- |
| General Consensus | N/A | While there is insufficient evidence to use MC in critically ill patients to alleviate suffering or PICS, MC has been shown to be relatively safe and so should be studied in critically ill patients as both an adjunct to, and harm reduction alternative to currently used medications such as opioids and benzodiazepines. THC content did not seem to be of significant concern for research in MC in critically ill patients. | N/A |
| 1 | Hoping and Caring | Unequivocally support MC research in all critically ill patients with a view to both alleviate acute and chronic ICU issues given that best practices/medications currently in use have been unable to ameliorate these complications. | “The hope is MC will help reduce the negative reaction patients have post ICU, post sedation and intubation and post the events that we do to patients daily. Patients should not have to deal with PTSD, anxiety, and fear when thinking back to their ICU stay. If we can attempt to even promote a more holistic and less stress inducing stay, why wouldn't we attempt it?” |
| 2 | Pragmatic Progress | Support MC research only in ventilated patients because they are concerned that MC use might lead to increased recreational use and harm and that there may be some unfounded ‘hype’ surrounding all of the potential for MC use. They see the involvement of cannabis-producing companies in research as a necessity and have neutral feelings about the impact of societal stigma surrounding research in medical cannabis. | “I feel ventilated patients would have the most PTSD, anxiety and pain. They would be least aware of what they are being treated with, therefore reducing the risk of long-term dependence.” |
| 3 | Cautious/Conservative and Protectionist | While supporting MC research in critically ill patients, more should be done to ensure adherence to best practices to minimize the need for more potentially harmful drugs being used to treat these patients. They are concerned about the involvement of cannabis-producing companies in research and want measures to ensure that the validity of the studies is not biased. Initially, MC research should not be conducted in vulnerable populations (children pregnant women), or in a manner that masks symptoms that patients may be experiencing. | “If the obtained data is influenced (by industry) in any way, the quality of the data will be affected.” |
