# Supplementary material for: Elicitation of stakeholder viewpoints about medical cannabis research for pain management in critically ill ventilated patients: A Q-methodology study
Source: PLoS One. 2021 Mar 18;16(3):e0248475. doi: 10.1371/journal.pone.0248475 (PMC7971896; doi:10.1371/journal.pone.0248475)
Supplement: Attachment — Submitted filename: PloS One Response to Editor.docx [file pone.0248475.s003.docx]

PloS One Response to Editor’s Comments/Questions

1) Sampling for q-methodology doesn’t require that study participants be representative of the entire population, but rather, that their viewpoints be representative of the entirety of viewpoints that may exist within the population (lines 143-145 and lines 305-307). The details of recruitment are described as non-probability sampling (lines 141-142) from healthcare providers and the lay public (lines 146-148). The sample size target was described in in lines 148-149. Critical care healthcare providers were recruited using email (lines 180-182), along with snowball sampling of other non-ICU healthcare providers (lines 182-185). Lay public was recruited using study brochures placed in the ICU (lines 185-188), along with approaching potential public participants in other areas of the hospital by volunteers (lines 188-193). There were no exclusion criteria and all participants had to provide informed consent prior to completing the q-study (lines 193-195). Response rate is not applicable measure in q-studies. The only demographic questions we asked of participants was whether they were healthcare providers or members of the lay public, and their age groups – these are described in Table 1 for phase 1 (lines 218-221) and Table 2 for phase 2 (lines 228-231).

2) We have qualified the general consensus statement by including consensus among sampled participants (line 285). We do comment on the limitations of the external validity of these results (lines 302), and the heterogeneity that may exist in the larger population (lines 310-313). However, we also qualify that q-methodology is robust to small sample sizes (lines 305-307) and the viewpoints that emerge from q-studies are generally accepted to adequately describe the complexity of viewpoints that exist among the larger population.
