# Supplementary material for: Elicitation of stakeholder viewpoints about medical cannabis research for pain management in critically ill ventilated patients: A Q-methodology study
Source: PLoS One. 2021 Mar 18;16(3):e0248475. doi: 10.1371/journal.pone.0248475 (PMC7971896; doi:10.1371/journal.pone.0248475)
Supplement: Attachment — Submitted filename: Response to Reviewers.docx [file pone.0248475.s004.docx]

PONE-D-20-16468
Elicitation of stakeholder viewpoints about medical cannabis research for pain management in critically ill ventilated patients: A Q-methodology study
PLOS ONE

Reviewer#1

Thank you for taking the time to review our manuscript.

1) Why did we choose Q-methodology over other qualitative techniques?

One of our group (GD) has a quantitative statistical background, and Q-methodology has a strong quantitative influence unlike the other qualitative methods. We reviewed several papers that used Q-methodology and we could not find a single example where the authors tried to justify their use of this technique versus using pure qualitative approaches as you’ve mentioned. We believe the reason is that Q-methodology is both a qualitative and quantitative approach to viewpoint solicitation and so is different enough from purely qualitative techniques that it would not be necessarily appropriate to discuss it only in terms of a qualitative approach.

2) Factor analysis question.

Q-methodology uses factor analysis to group overall patterns of statement rankings about a topic. It is the common viewpoints and opinions that emerge that are uncovered by the factor analysis, whereas traditional factor analysis looks for correlations between characteristics such as how responses to different items in a questionnaire can be grouped together.

3) Measurement invariance

We are not comparing factors across any groups; we are looking for patterns in the overall ranking of statements. Traditional factor analysis is not used in Q-methodology, so measurement invariance is not part of the quantitative analysis. We did provide a reference (15) explaining the quantitative approach to Q-methodology that we feel help clarify the approach for those who are less familiar with this method.

4) Final factor labelling

In Q-methodology, the factor labelling is the final step and the product of the technique. We cannot move it forward as it emerges after the quantitative analysis and a review of all the qualitative statements supporting the ranking patterns that emerged as a result of the quantitative analysis.

5) Method limitation

We did provide additional limitations beyond that described by the reviewer that included the imbalance in the first phase between the lay public and healthcare providers, and also that the final 3 viewpoints that emerged did not include the patterns elicited in 32% of the remaining participants. While we have used the term ‘convenience’ sample, it is not to be interpreted in the context of survey sampling as Q-methodology is not a survey technique and is not limited by sample representativeness. Instead, Q-methodology seeks to ensure that sufficient stakeholders are included whose viewpoints should span the imaginable viewpoints that might exist on any topic of interest. The ‘convenience’ part of our sample was that we solicited these viewpoints from stakeholders all located from the same hospital. This would not be considered to be a limitation in Q-methodology.

Reviewer#2

Thank you for taking the time to review our manuscript

1) Introduction too long

We wanted to ensure we provided sufficient background for the Q-methodology approach as we recognize this method is unfamiliar to many readers. We do feel that it is comprehensive in both providing the background on analgosedation in critically ill patients, cannabis use and Q-methodology in a way that provides the reader with enough information to understand the design and results.

2) Appendix materials

The output of q-methodology is quite extensive and sometimes difficult to understand for unfamiliar readers, so we decided to be as comprehensive as possible and include almost all of it both the results and the appendix. We felt we had to use an extensive appendix to support the qualitative portions of the method.

3) Inferential statements in results lines 238, 242, 245

These were removed and replaced in discussion

4) Guidance on important aspects related to cannabis researchers

We have updated that section of the discussion to highlight some important inputs into design, conduct, analysis and reporting commented on by the stakeholders in their subjective statements.

5) Conclusion

We have modified the conclusion to better reflect the reviewer’s comments
