# Supplementary material for: Elicitation of stakeholder viewpoints about medical cannabis research for pain management in critically ill ventilated patients: A Q-methodology study
Source: PLoS One. 2021 Mar 18;16(3):e0248475. doi: 10.1371/journal.pone.0248475 (PMC7971896; doi:10.1371/journal.pone.0248475)
Supplement: Attachment — Submitted filename: renamed_89195.docx [file pone.0248475.s005.docx]

Reviewer’s comments: Elicitation of stakeholder viewpoints about medical cannabis research for pain management in critically ill ventilated patients: A Q-methodology study

Thanks for the opprtunity to review the manusript. The study method utilized seems somewhat novel and concentrates on an interesting area that needs exploration.

However, the study is challenged by significant methodological issues.

Utilizing a strategic sampling method and using an “informal approach” to finalize the “statements” included in the q- test indicates that the statement's selection was subjective to bias. The authors should mention this limitation in the discussion part and also they should comment on what basis the authors strategically choose the survey participants?

The authors should also describe the statements that were chosen for the survey. The authors should mention how they excluded the possibility of the same person being examined in both phases of the survey. The possibility of double-counting increases as they have utilized a snowballing technique to find the study participants.

There is a certain lack of clarity about the abbreviations and designations used by the authors. For example, at first, the authors have mentioned that phase 1 q set was distributed to all “intensive care unit staff” with snowball sampling of other hospital staff. They also mentioned that the face 2 q simple survey was “similarly distributed”. However, later on in the results section and on the tables, the authors have changed the terminology of "intensive care unit staff" with "healthcare providers". We need to keep in mind that staff members can be used to define various designations in a healthcare system, whereas providers are used only for doctors, nurse practitioners, and other very limited sets of professionals. The perspectives of **healthcare providers** can be very different from that of a layperson. Clubbing the healthcare providers and laypersons in the same group and asking for their opinion on a scientific research topic (whether medical cannabis research should be carried out or not and concluding on whom should be included in those studies) seems to be flawed. Healthcare providers and laypersons have wide differences in their knowledge and expertise in research and knowledge about cannabis.

A better study design could have been a comparison between healthcare providers viewpoint with that of laypersons (the authors may not agree, but this is the reviewer’s viewpoint). Similarly, it is hard to find if the authors have mentioned the full form of the abbreviation used in the tablets such as HCP ICU+, HCP ICU-, or Public ICU+ and Public ICU -.

The authors also should elaborate on factor 1, factor 2 factor 3 other than simply saying “hoping and caring”, “pragmatic progress” and “cautious/conservative and protectionist”.

The authors should also define what they mean by the “stakeholders” of medical cannabis research. This is particularly important because, the stakeholders of cannabis research does not only include the intensive care providers and family members but also includes, a wide range of other individuals such as government agencies, pain medicine specialists, substance users, psychiatrist and behavioral specialist that the authors have not included in this survey. The authors seem to have used the term stakeholders of medical cannabis research in a very simplistic way.

The authors also mentioned that the results of the study also provided guidance on "whom to include and exclude in medical cannabis research" and "what outcome should be targeted for impact" and "who should be involved in the design, financing, conduct an analysis of the research". Unfortunately, this study does not seem to really conclude the above based on the study method hat the authors have used. Can the author comment on how they concluded the above crucial issues based on a survey involving the “viewpoint (which may or may not have any scientific basis)” of laypersons and ICU providers? The authors should also comment on how did this survey help them to come to a conclusion that "at-risk populations such as pregnant women, those with substance abuse disorders or mental health patients were excluded....." Can a scientic conclusion be based on a survey involving a significant number of laypersons and professionals who may or may not have a good knowledge about the scientific evidence base on the research question? Can we really come to a concluding statement on inclusion and exclusion criteria of cannabis research by a survey?

A significant part of the conclusions drawn by the authors seems to lack any scientific basis. The authors concluded that “given this stakeholders input, we believe there is sufficient support for proceeding with medical cannabis research as an adjunct to analgosedation in critically ill ventilated patients”. This statement seems to lack sufficient ground because the decision to proceed with medical research does not depend upon the lay person’s understanding or viewpoint on a scientific matter but on the basis of the level of evidence and other ethical and operational issues. The authors also conclude that the next step in the research is to determine the correct dosages of medical marijuana also do not seem to be the next step following this survey.
