# Supplementary material for: Elicitation of stakeholder viewpoints about medical cannabis research for pain management in critically ill ventilated patients: A Q-methodology study
Source: PLoS One. 2021 Mar 18;16(3):e0248475. doi: 10.1371/journal.pone.0248475 (PMC7971896; doi:10.1371/journal.pone.0248475)
Supplement: Attachment — Submitted filename: Response to Reviewers.docx [file pone.0248475.s006.docx]

Table of Contents

[PONE-D-20-16468R1	2](#_Toc63766832)

[Editor	2](#_Toc63766833)

[Q1	2](#_Toc63766834)

[Reviewer#3	2](#_Toc63766835)

[Q1	2](#_Toc63766836)

[Q2	3](#_Toc63766837)

[Q3	3](#_Toc63766838)

[Q4	3](#_Toc63766839)

[Q5	3](#_Toc63766840)

[Q6	3](#_Toc63766841)

[Reviewer#4	4](#_Toc63766842)

[PONE-D-20-16468	5](#_Toc63766843)

[Reviewer#1	5](#_Toc63766844)

[Q1	5](#_Toc63766845)

[Q2	5](#_Toc63766846)

[Q3	5](#_Toc63766847)

[Q4	5](#_Toc63766848)

[Q5	5](#_Toc63766849)

[Reviewer#2	6](#_Toc63766850)

[Q1	6](#_Toc63766851)

[Q2	6](#_Toc63766852)

[Q3	6](#_Toc63766853)

[Q4	6](#_Toc63766854)

[Q5	6](#_Toc63766855)

PONE-D-20-16468R1
Elicitation of stakeholder viewpoints about medical cannabis research for pain management in critically ill ventilated patients: A Q-methodology study
PLOS ONE

## Editor

Thank you for taking the time to review our manuscript

Q1) How generalizable are our conclusions given the sample was derived from a single hospital site?

We specifically chose a Q-methodologic approach because of the limits on sampling from a single hospital site. Unlike other survey techniques that require random sampling to derive a valid representation of the population, Q-methodology only requires that the sample of viewpoints generated (q-set) from the participants (and other sources) to the topic statement represent the theoretic population of all viewpoints. The subsequent ranking of these viewpoints might certainly differ between populations as we discussed in the limitations (lines 345-353), but the underlying theory supporting this model suggests that human beings are much more similar than they are different. There have been q-methodology studies where there was a single participant who provided meaningful and generalizable insights about a topic. As we explained in our Q-methodology design section, the sample sizes needed for this study design are minimal, rarely ever exceeding 40 patients in the literature. The advantage of having used this approach is that we can simply re-administer the q-sort to different populations as part of a qualitative arm of a randomized controlled trial exploring the impact of medical cannabis on analgosedation in critically ill patients. This would provide us some insight on both issues of recruiting and acceptability at different participating test sites.

## Reviewer#3

Q1) Strategic sampling of q-statements and participants

In every q-methodology design, there is always an ‘informal’ approach to the selection of the final q-statements from the q-set generated in phase 1. We have included several statements in the limitations section to reflect this intrinsic aspect of every q-study (lines 336-345). As for participant sampling, we wanted to sample participants who might be direct stakeholders in any subsequent study involving medical cannabis in critically ill patients. That was the strategic component of our sampling (lines 140 to 141). Our sampling technique is consistent with that recommended for all q-studies. As for participants completing both phase 1 and phase 2, we specifically asked participants to complete only one or the other, but even if they completed both phases, there is nothing intrinsically wrong with this in q-studies. Many q-studies use the same participants for both phases as there is no strong methodologic reasons for prohibiting this and it is much more efficient.

Q2) Selection of q-sort statements

We have included more detail about the *informal process* used in this study to select the final q-sort statements (lines 156 to 169). The final q-sort statements have been included in the supplement Table 1 (lines 168-169). We have also included a statement in the limitations section about the possible selection bias that is intrinsic to q-studies regarding the final inclusion of q-sort statements (lines 336-344).

Q3) Healthcare providers and lay public nomenclature

We have defined what we mean by these terms and abbreviations used in Table 1 (lines 239 to 244). We did describe the differences in factor loadings between healthcare providers and lay people, including differences between age groups (lines 272 to 282).

Q4) Stakeholders definition

We have included the definition of our use of the term stakeholder in this study (lines 140 to 141).

Q5) Conclusions from viewpoints about subsequent study design elements

In phase 2, each participant was asked to provide qualitative supporting statements about why they had ranked q-sort statements at either extreme end, neutrally and their overall impression of the topic statement. Needless to say, there were many hundreds of these qualitative statements that we could not practically include in the paper. These qualitative statements are an integral part of the q-study conclusions and the reason q-studies are referred to as mixed methods. Many of the conclusions we include are informed by the content of these qualitative statements as much as they are informed by the quantitative factor analysis. As you might imagine, the most difficult part of any q-study is to integrate the factor analysis and the qualitative statements into summary statements as we have done. As for your question about the public participating in research design, this is already happening globally (James Lind Alliance <https://www.jla.nihr.ac.uk/>, patient-centered outcomes research institute <https://www.pcori.org/> to name just a few) and is considered to be the ideal. How we include the public in guiding research can be done in many ways. Whether they sit on research committees or they participate in other ways such as having their opinions or viewpoints know through surveys or other methods seems to be less important than ensuring their opinions and viewpoints are acknowledged and used to help inform research that is patient centered. Our study represents a novel way to include stakeholders and the public into the research planning process.

Q6) Next steps for research based on our study

We sought to answer the following questions: would medical cannabis research be acceptable to stakeholders directly affected by its use in critically patients, and what design considerations should we consider. We feel this q-study helped answer those questions. We do believe a pilot study assessing medical cannabis dosing and safety and feasibility of recruitment are the next steps for medical cannabis research in the critically ill.

## Reviewer#4

There did not appear to be any follow-up questions from this reviewer.

PONE-D-20-16468
Elicitation of stakeholder viewpoints about medical cannabis research for pain management in critically ill ventilated patients: A Q-methodology study
PLOS ONE

## Reviewer#1

Thank you for taking the time to review our manuscript.

Q1) Why did we choose Q-methodology over other qualitative techniques?

One of our group (GD) has a quantitative statistical background, and Q-methodology has a strong quantitative influence unlike the other qualitative methods. We reviewed several papers that used Q-methodology and we could not find a single example where the authors tried to justify their use of this technique versus using pure qualitative approaches as you’ve mentioned. We believe the reason is that Q-methodology is both a qualitative and quantitative approach to viewpoint solicitation and so is different enough from purely qualitative techniques that it would not be necessarily appropriate to discuss it only in terms of a qualitative approach.

Q2) Factor analysis question.

Q-methodology uses factor analysis to group overall patterns of statement rankings about a topic. It is the common viewpoints and opinions that emerge that are uncovered by the factor analysis, whereas traditional factor analysis looks for correlations between characteristics such as how responses to different items in a questionnaire can be grouped together.

Q3) Measurement invariance

We are not comparing factors across any groups; we are looking for patterns in the overall ranking of statements. Traditional factor analysis is not used in Q-methodology, so measurement invariance is not part of the quantitative analysis. We did provide a reference (15) explaining the quantitative approach to Q-methodology that we feel help clarify the approach for those who are less familiar with this method.

Q4) Final factor labelling

In Q-methodology, the factor labelling is the final step and the product of the technique. We cannot move it forward as it emerges after the quantitative analysis and a review of all the qualitative statements supporting the ranking patterns that emerged as a result of the quantitative analysis.

Q5) Method limitation

We did provide additional limitations beyond that described by the reviewer that included the imbalance in the first phase between the lay public and healthcare providers, and also that the final 3 viewpoints that emerged did not include the patterns elicited in 32% of the remaining participants. While we have used the term ‘convenience’ sample, it is not to be interpreted in the context of survey sampling as Q-methodology is not a survey technique and is not limited by sample representativeness. Instead, Q-methodology seeks to ensure that sufficient stakeholders are included whose viewpoints should span the imaginable viewpoints that might exist on any topic of interest. The ‘convenience’ part of our sample was that we solicited these viewpoints from stakeholders all located from the same hospital. This would not be considered to be a limitation in Q-methodology.

## Reviewer#2

Thank you for taking the time to review our manuscript

Q1) Introduction too long

We wanted to ensure we provided sufficient background for the Q-methodology approach as we recognize this method is unfamiliar to many readers. We do feel that it is comprehensive in both providing the background on analgosedation in critically ill patients, cannabis use and Q-methodology in a way that provides the reader with enough information to understand the design and results.

Q2) Appendix materials

The output of q-methodology is quite extensive and sometimes difficult to understand for unfamiliar readers, so we decided to be as comprehensive as possible and include almost all of it both in the results and the appendix. We felt we had to use an extensive appendix to support the qualitative portions of the method.

Q3) Inferential statements in results lines 238, 242, 245

These were removed and replaced in discussion

Q4) Guidance on important aspects related to cannabis researchers

We have updated that section of the discussion to highlight some important inputs into design, conduct, analysis and reporting commented on by the stakeholders in their subjective statements.

Q5) Conclusion

We have modified the conclusion to better reflect the reviewer’s comments
